# Effects of cow efficiency phenotype on performance, energy partitioning, ultrasound carcass composition, and gas exchange during gestation and lactation

**DOI:** 10.1093/tas/txag021

**Published:** 2026-02-25

**Authors:** Mariana E Garcia-Ascolani, Mikayla Moore, Emma Briggs, Paul Beck, Eric DeVuyst, David Lalman

**Affiliations:** Department of Food and Animal Sciences, Oklahoma State University, Stillwater, OK, 74078, United States; Department of Food and Animal Sciences, Oklahoma State University, Stillwater, OK, 74078, United States; Department of Food and Animal Sciences, Oklahoma State University, Stillwater, OK, 74078, United States; Department of Agricultural Economics, Oklahoma State University, Stillwater, OK, 74078, United States; Department of Food and Animal Sciences, Oklahoma State University, Stillwater, OK, 74078, United States

**Keywords:** beef cow, feed efficiency, methane, phenotype, residual net energy, residual feed intake

## Abstract

This study investigated the effects of residual feed intake (RFI) and residual net energy recovered (RNE) classifications on the performance, carcass composition, milk production, and gas exchange of crossbred Angus beef cows consuming an unprocessed forage diet during gestation and lactation. Thirty-six multiparous fall-calving cows were monitored for feed intake, body weight, body condition, ultrasound-based carcass traits, milk yield and quality, carbon dioxide and methane emissions, and oxygen consumption using automated feeding and gas measurement systems. Cows were retrospectively classified as efficient, moderate, or inefficient for RFI and RNE during each physiological stage. RFI-efficient cows consistently consumed less forage, both in absolute terms and as a percentage of body weight during gestation (both, *P* < 0.01) and lactation (both, *P* ≤ 0.03). Divergence in RFI did not have negative effects on body weight, average daily gain (ADG), body condition score or carcass composition at gestation (*P* ≥ 0.13) or lactation (*P* ≥ 0.13). However, RFI classification significantly affected allocation of energy to maintenance in gestating (*P* < 0.01) and lactating (*P* = 0.02) cows, with inefficient cows allocating more metabolizable energy to maintenance (MEm) compared to moderate and efficient cows. RNE-efficient cows had greater ADG during gestation (*P* < 0.01) and had a similar tendency in lactation (*P* = 0.10), allocating more net energy to tissue accretion (*P* ≤ 0.05, both stages). Neither RFI nor RNE classification affected milk yield or composition (*P* ≥ 0.18), except for RNE-efficient cows having greater milk lactose concentration (*P* = 0.01) than RNE-moderate cows. Methane emissions per kilogram of DMI were greater in RFI-efficient cows during gestation (*P* < 0.01) but not during lactation (*P* = 0.22). Notably, while feed intake was more persistent across stage of production (*r =* 0.66), efficiency classifications were not stable across physiological stages (*r =* 0.33 and 0.25 for RFI and RNE, respectively). In addition, there was a moderate relationship between RFI and RNE at gestation (*r =* 0.36) and weak relationship during lactation (*r =* 0.24). These findings suggest that RFI and RNE are complementary but distinct measures of efficiency, and that selection for either trait can improve feed efficiency and a more targeted achievement of objectives in beef cow herds without compromising productivity or maternal performance.

## Teaser

These results suggest that intake and maintenance requirements exhibit greater persistence across physiological stages, whereas efficiency rankings are less stable. This suggests the need to assess forage-use efficiency within biologically relevant phases of the cow-calf production cycle rather than assuming a single, stable measure of lifetime efficiency.

## Introduction

In recent decades, there has been a growing interest in measuring feed efficiency in cattle. The aim of evaluating, reporting, and utilizing feed efficiency parameters in cattle selection is to produce improved production traits while maintaining or reducing input costs. This approach contributes to the overall sustainability of beef production (Ojo et al. 2024). Feed efficiency was primordially defined as the gain in body weight (BW) resulting from the consumption of a given amount of feed or its inverse (Koch et al. 1963). However, efficiency of feed use is not a directly measurable trait, and variations in efficiency may arise due to differing environmental conditions or the various energy requirements of the animals. Koch et al. (1963) proposed using residual feed intake (RFI) as a measure of efficiency. Residual feed intake is a complex trait used to phenotype cattle. It measures the variation in feed intake independent of BW or growth rate and is calculated by taking the difference between the actual recorded feed intake and the predicted feed intake within a contemporary group’s specific physiological stage, maintenance needs, or production level (Black et al. 2013). A cow’s efficiency in metabolizing consumed energy and directing it to various sinks (maintenance, maternal tissue, milk production, and fetal development) is influenced by both genetic and phenotypic traits. Residual feed intake is independent of growth rate and mature body weight, allowing selection for more efficient animals without increasing mature body weight. This is particularly beneficial for the cow herd, as maintenance requirements account for a large proportion of total feed costs. Therefore, permanent and cumulative savings could be made over the cow’s lifetime (Fitzsimons et al. 2014). However, a caveat of measuring feed efficiency is that it requires assessing individual intake, which is particularly challenging for grazing cows; consequently, there is very limited information available on residual feed intake (RFI) or feed intake measurements in grazing systems (Meyer et al. 2008; Black et al. 2013; Lawrence et al. 2013; Fitzsimons et al. 2014). In addition, differences in RFI among grazing animals are largely influenced by their feeding behavior, which affects ruminal fermentation and eating activity, especially with forage diets (Fitzsimons et al. 2014). Moreover, lower intake rates and slower rumen passage rates limit voluntary feed intake, potentially reducing the inherent dry matter intake (DMI) potential of the cow. This may account for some unexplained variation in RFI, resulting in a lower *R*^2^ value for DMI prediction compared to growing animals consuming an energy-dense diet (Fitzsimons et al. 2014; Kenny et al. 2018). More recently, residual average daily gain (RADG) has been proposed as another tool to measure efficiency (Northcutt and Bowman, 2010). Residual ADG is calculated by subtracting the predicted ADG from observed ADG. Therefore, a positive or high RADG value is desired as it indicates more gain per unit of feed consumed. Applying RADG to feedlot animals should improve feed efficiency during the finishing phase. However, this selection approach may pose challenges for cow-calf producers (Ojo et al. 2024) since selecting based on RADG may result in larger, heavier cows with higher nutrient requirements (Faulkner 2016), which is not advantageous for cow efficiency or the sustainability of the production system. In addition, reliance on gain as a key metric in many efficiency systems limits its applicability to mature, non-growing cows where revenue is generated from selling their calves (Delver et al. 2023). A more appropriate way to phenotype mature beef cows would be by classifying them by the actual energy allocated to support body maintenance functions (such as basal metabolism, voluntary activity, thermal regulation and product formation, as described by Ferrell and Oltjen, 2008) and to tissue accretion, milk production, conceptus, among others. We propose to use residual net energy recovered (RNE) calculated by subtracting the predicted net energy retained from the observed net energy retained (accounted as the sum of the net energy allocated to conceptus and maternal tissue accretion, plus milk energy during lactation) in gestating and lactating cows. There is limited data investigating the relationship between feed and energy efficiency and beef cows’ performance and maternal traits at gestation (Fitzsimons et al. 2014; Holder et al. 2022) and lactation (Black et al. 2013; Lawrence et al. 2013; Sprinkle et al. 2021).This lack of data is partly a consequence of most performance traits being measured on growing beef cattle consuming high-concentrate diets and the lack of methods to accurately measure individual DMI in beef cows. This is significant, especially considering that approximately 59% of the total feed consumption required to finish 1 kg of carcass weight comes from grazed forage (82% if harvested forage is also included in the calculation, Rotz et al. 2019). To help bridge this knowledge gap, a two-phase experiment using gestating and lactating beef cows was conducted to determine how the retrospective ranking of cows based on RFI and RNE influences different performance and maternal traits. Therefore, beef cows were offered an unprocessed forage diet with minimal supplementation, and their performance, carcass composition, and gas exchange parameters were measured at gestation and lactation.

## Materials and methods

The Oklahoma State University (OSU) Institutional and Animal Care and Use Committee (IACUC) approved all procedures involving the use of animals (IACUC Protocol 21-12). A two-phase experiment evaluating various parameters in beef cows at gestation and subsequent lactation was conducted at OSU’s Kenneth and Caroline Eng Pens at the Range Cow Research Center, North Range Unit near Stillwater, OK.

### Animals and management

Data were obtained from a contemporary group of 36 multiparous fall-calving Angus and Angus × Hereford gestating beef cows (573 ± 95 kg BW; 104 ± 20 d pregnant). The study lasted for 64 d, which included a 10-d adaptation period to the diet and feeding conditions, followed by a 54-d data collection period. The calves were weaned 17 d before the beginning of the adaptation period. Cows were fitted with radiofrequency identification (RFID) tags on their right ear and were randomly assigned to five dry lot pens with shade cloths and windbreaks on the north and south perimeters. Each pen was equipped with two SmartFeed individual intake units (SmartFeed, C-Lock Inc., Rapid City, SD). Cows were provided with ad libitum access to long-stem, unprocessed bermudagrass hay (8.7% crude protein (CP) and 59% total digestible nutrients (TDN)) and mineral mix along with 1 kg/d of concentrate pellets (14% CP, 75% TDN). To account for potential waste, prior to feeding, each round bale of hay was weighed on an electronic scale. All feeders were cleaned out weekly or after a rain event and recalibrated according to manufacturer instructions. Orts and hay accumulated around the feeders were weighed weekly at the time of feeder cleanout (Holder et al. 2022). Recovered hay (cumulative hay disappearance indicated by the intake system plus orts) was > 90% of the original bale weight in each of the weekly intervals. Feed bins were checked and replenished daily at 0700 to ensure continuous availability of hay and to prevent empty bunks. Additionally, hay was replaced after heavy rain events to maintain quality and access. This management approach ensured that feed was never restricted and that all cows had unlimited access to forage at all times. At the end of the 54-d collection period, cows were relocated to a bermudagrass pasture where they grazed during late gestation and early lactation. Beginning 125 ± 19 days after calving, cows were moved into the same feed intake facility. Twenty-six cows from the original group were studied (560 ± 48 kg BW; 58 ± 1 d pregnant). The trial included 14 d of adaptation and 70 d of data collection, conducted under conditions similar to those previously described. As in the gestation trial, cows had unrestricted access to long-stem unprocessed bermudagrass hay (8.3% CP, 54% TDN) and mineral mix plus 1 kg/d of a concentrate pellet (20% CP, 75% TDN).

### Feed intake and nutrient apparent total tract digestibility

Daily feed intake was measured using the SmartFeed system, which combines RFID technology with a load cell and feed bin to continuously log data to monitor and record the feed intake per visit for each animal. Each SmartFeed unit transmits data independently via a Wi-Fi network to a cloud-based server. When an animal approached the feed bin, the system identified the RFID and recorded the animal number, feeder number, start and end time of feeding, duration of the event, initial and final weight of the hay in the bin and the quantity of hay removed during a visit. Raw intake data were downloaded from the C-Lock Inc. portal using an automated R script, which authenticated via application Programming Interface (API, developed by Brennan et al. 2024) and extracted visit-level intake records for selected equipment IDs within the desired date range. The data were imported into R and merged with reference files to append animal identification (RFID and animal number) and removed. All data processing, including data cleaning, outlier removal, and summary table generation, was performed using R (version 4.4.2). Hay samples were obtained from individual feed bunks at three depths to ensure a representative sample. To minimize disruption of natural feeding behavior and avoid the potential effects on performance associated with the use of external markers and repeated fecal collections (confining animals to chutes for measurements of total fecal output, marker concentration, and digestibility at discrete time points rather than reflecting true daily intake) the authors opted to offer feed in a dry lot system and to collect feed and fecal samples following the schedule of body weight measurements. Fecal grab samples were collected only on days when cows were already being moved to the chute for body weight measurements and were composited by cow across sampling days, so no additional handling events beyond routine weighing were required for digestibility determination. This approach allowed for more accurate and consistent measurement of individual intake while maintaining nutritional conditions similar to those experienced by grazing cows, without the additional stressors and methodological constraints inherent to marker-based intake estimation. Fecal samples were collected directly from the rectum of individual cows. Hay and fecal samples were dried at 55°C for 72 h (or until a constant weight was reached), ground to pass a 2‑mm screen using a Wiley mill (Thomas Scientific, Swedesboro, NJ) and composited by phase (feed) or by cow within phase (fecal samples). Apparent total tract nutrient digestibility was estimated using acid detergent insoluble ash as an internal marker as described by Cochran and Galyean (1994) and Kanani et al. (2014).

### Body weight, body condition score and carcass ultrasound

Cows were weighed on two consecutive days at the beginning and end of each collection period and every 7-d for the duration of each experiment. Initial BW, final BW, and ADG were computed for each cow by regressing BW on day of study (Ferrell and Jenkins 1984). Body condition scores (BCS, scale = 1 to 9; Wagner et al. 1988) were assigned at the initiation, midpoint and termination of each phase by two trained personnel. The scores from both personnel were averaged to give each cow one score per timepoint. Cows were ultrasonically scanned at the beginning and end of each phase by trained personnel. A portable convex sector/linear scanner (Aloka 500-V, Corometrics Medical Systems, Wallingford, CT) was used to measure the area of the longissimus dorsi muscle (ribeye area, **REA**) and the depth of backfat (BF) at the 13th thoracic rib, as well as the depth of rump fat.

### Milk production and nutritional composition

During the lactation phase, milk yield and composition were assessed four times throughout the trial, on d 8, 22, 43 and 64. The milking procedure was adapted from Marston et al. (1992). Briefly, at 0800 h on the day before milk collection, calves were separated from their dams to prevent suckling until 1200 h. At that time, the pairs were reunited and allowed to nurse until calves were satiated (no longer than 45 min), after which they were separated from their damns again. At 2000 h, the milking process began. Approximately 3 min before milking, 2 mL of oxytocin were injected intramuscularly to facilitate milk let-down. The udders were cleaned with a pre-dip solution and patted dry. A portable milking machine was then used to collect the milk until the flow stopped. After removing the milker, each quarter was stripped by hand to ensure complete evacuation of the udder. The milk collected from the machine and the hand-stripped milk were combined, weighed, and a 50-mL sample, containing 2-bromo-2-nitropropane-1,3-diol, was shipped to an external laboratory for analysis of nutritional composition. After milking, an iodine-containing solution was used to dip each teat, and the cows were returned to their pen to be reunited with their calves.

### Gas exchange measurements

A GreenFeed (C-Lock Inc., Rapid City, SD) gas emission monitoring (GEM) system was used to determine daily carbon dioxide (CO_2_) and methane (CH_4_) emissions, as well as daily oxygen (O_2_) consumption in cows. Briefly, the GreenFeed system is an automated head-chamber device that continuously measures gas concentrations in the animal’s exhaled breath. The system uses a proximity sensor and RFID technology to detect when an animal approaches and positions its head within the chamber. Upon detection, the system dispenses a small, predetermined amount of feed pellets (bait) from an overhead hopper to encourage the animal to remain in the chamber for approximately 3 to 7 min. During each visit, the system collects spot measurements of the gases in the animal’s breath. By aggregating data from multiple visits throughout the day, the GreenFeed system calculates average daily emission of CO_2_ and CH_4_ and consumption rate of O_2_. As described in previous publications from our group (Holder et al. 2022), the GEM was rotated through the pens after adaptation to the diet in each phase, spending no less than nine consecutive days in each pen. The daily collection protocol was restricted to four visits to the GEM per cow within a 24-h period, with a minimum interval of 4 h between visits to promote a diurnal usage pattern. As recommended by C-Lock Inc., only cows having at least 20 gas emissions records lasting a minimum of 3 min per record were included in the final data set. Raw data were uploaded from the GreenFeed system to the C-Lock Inc. server via Wi-Fi, where they were processed using proprietary algorithms to generate daily average emission values per cow. The processed data, including daily means for each animal, were then reported to users for further analysis. Heat production (HP) was calculated from gas exchange using a simplified CO_2_− and respiratory quotient (RQ)‑based equation derived from the Brouwer (1965) calorimetry framework, as presented by Kaufmann et al. (2011) and applied by Pereira et al. (2015). Specifically, HP (MJ/d) was computed as:


HP=[4.96+(16.07/RQ)]×CO2/1,000

Where ${\text{CO}}_{2}$ is carbon dioxide production (L/d) and $\text{RQ}={\text{CO}}_{2}/O_{2}$. In this formulation, the coefficients for $O_{2}$ and ${\text{CO}}_{2}$ were re-estimated under the assumption of a fixed contribution of protein oxidation, so an explicit urinary N term is not included. This approach avoids the logistical and animal-welfare challenges of repeated urine collection and has been shown to have minimal impact on HP and downstream estimates of dietary NEm and NEg under typical conditions (Proctor et al. 2024), although it implicitly assumes relatively stable protein oxidation and may slightly underestimate HP in situations with unusually high protein turnover.

### Computation of residual feed intake (RFI) and residual net energy (RNE)

Residual feed intake (RFI) and residual net energy recovered (RNE) were calculated using a multi-step modeling approach (PROC GLMSELECT and PROC MIXED; SAS Inst. Inc., Cary, NC). Model selection was performed by comparing candidate models using the coefficient of determination (*R*^2^) and selecting the most parsimonious model with the potential for broader applicability to other datasets. For the determination of RFI in gestating cows, the classical model proposed by Koch et al. (1963), which regresses observed DMI against ADG and mid-test metabolic BW (MTBW^0.75^) was finally selected (*R*^2^ = 0.28). The final RFI model (Table 1) was:


$${\mathrm{DMI}}_{p}=\beta_{0}+\beta_{1}ADG+\beta_{2}{\mathrm{MTBW}}^{0.75}+\varepsilon$$

**Table 1:** Regression coefficients and model fit (*R*^2^) for predicting DMI^1^and NEr^2^ in gestating and lactating beef cows.

Predicted trait	Physiological stage	Regression coefficients^3^					Model fit (*R*^2^)
		Intercept	ADG	DMI	MTBW^0.75^	ECM	
**DMI**	Gestation	1.59	3.26		0.08		0.28
	Lactation	2.04	1.02		0.08	1.05	0.62
**NEr**	Gestation	51.29		2.49	−0.49		0.29
	Lactation	55.18		4.04	−0.63		0.53

1 DMI, dry matter intake.

2 NEr, total net energy recovered.

3 ADG, average daily gain; MTBW^0.75^, mid-test metabolic body weight; ECM, energy corrected milk yield.

Where ${\mathrm{DMI}}_{p}$ is the individual daily DMI predicted. For the calculation of RNE in gestating cows, net energy retained (NEr) was regressed against DMI and MTBW^0.75^. This base model demonstrated adequate predictive performance (*R*^2^ = 0.28). The final RNE model was:


$${\mathrm{NEr}}_{p}=\beta_{0}+\beta_{1}DMI+\beta_{2}{\mathrm{MTBW}}^{0.75}+\varepsilon$$

Where ${\mathrm{NEr}}_{p}\;$is the individual net energy retained predicted.

Model selection for predicting DMI in lactating cows was conducted by comparing the model of Koch et al. (1963; *R*^2^ = 0.28) with alternative models incorporating additional predictors. Model fit improved substantially (*R*^2^ = 0.62) when energy corrected milk (ECM) was included in the regression. The final model to predict DMI in lactating cows was:


$${\mathrm{DMI}}_{p}=\beta_{0}+\beta_{1}ADG+\beta_{2}{\mathrm{MTBW}}^{0.75}+\beta_{3}ECM+\varepsilon$$

The following model (*R*^2^ = 0.53) was selected for the prediction of RNE in lactating cows:


$${\mathrm{NEr}}_{p}=\beta_{0}+\beta_{1}DMI+\beta_{2}{\mathrm{MTBW}}^{0.75}+\;\varepsilon$$

For both gestating and lactating cows, residuals for DMI and NEr were calculated as the difference between observed and model-predicted values. Residual feed intake represents the residual from the DMI model, while RNE is the residual from the NEr model.

Cows were retrospectively grouped into three efficiency categories (efficient, moderate, and inefficient) for both RFI and RNE using k-means clustering (PROC FASTCLUS; SAS Institute Inc., Cary, NC, USA). This unsupervised, data-driven approach classified individual animals according to their RFI and RNE values in each experimental phase.

### Calculation of energy allocation

For gestating beef cows, total net energy retained (NEr) was determined as the sum of net energy partitioned into the tissues of the gravid uterus (NEy) and maternal tissue (NEt). Energy expenditure for maintenance was calculated on a metabolizable energy base (MEm). Energy accretion in the tissues of the gravid uterus was determined for each day of the collection period based on the NASEM (2016) formula:


$$\mathrm{NEy}\;(\mathrm{Mcal}/d)=[\mathrm{CBW}\times {(0.05855-0.0000996\times DP)\times e}^{\left(0.03233\times DP-0.0000275\times {DP}^{2}\right)}]/1000$$

Where DP is day of pregnancy and CBW is calf birth BW. The final NEy for each individual cow was calculated as the average NEy across the collection period. For the calculation of NEy, cow BW was adjusted by subtracting the estimated weight of the fetus and associated uterine tissue from BW at the start and end of the test period (Fitzsimons et al. 2014). The weights of the products of conception at the start and end of the test period were calculated using the NRC (2000) equation as follows:


$$\mathrm{Conceptus}\;(kg)=[\mathrm{Calf}\;\mathrm{birth}\;BW\;(kg)\times 0.01828]\times e^{\left[(0.02t)-(0.0000143t/t)\right]}$$

Where t is the day of gestation. The conceptus-adjusted BW was then used to calculate conceptus-adjusted mean BW at the beginning and end of the collection period. Shrunk BW was calculated as described in Chizzotti et al. (2019) and empty BW was calculated as described in NASEM (2016). Energy directed to maternal tissue was computed as described in NASEM (2016) and based on equations first published by NRC (1996, 2000). Briefly, two regression equations were built using tabular values of BCS as a predictor of net energy gain or loss per unit of change in empty BW. Then, individual average BCS and empty BW change of each cow were fit into the appropriate regression equation (based on gain or loss of empty BW during the collection period) to derive NEt.

Metabolizable energy intake (MEI) was derived from subtracting urinary and methane energy from digestible energy intake. Urinary energy was calculated based on estimations of urinary nitrogen derived from tabular regressions including nitrogen and DMI intake as predictors as described by Waldrip et al. (2013) and multiplying urinary nitrogen (g/d) by 0.0146 (Morris et al. 2021). Energy lost in the form of methane was calculated applying the Ideal Gas Law and using individual average methane emission recorded during the collection period. Metabolizable energy for pregnancy was determined based on the following equation (NASEM 2016).


MEy=NEy/0.13

Whereas MEt was determined by dividing NEt by the partial efficiency of use of metabolizable energy (ME) to net energy ($k_{m})$ from the equation of Garrett (1980) as follows:


$$k_{m}=(1.37\;ME-0.138{ME}^{2}+0.0105{ME}^{3}-1.12)/ME).$$

Metabolizable energy for maintenance was determined as:


MEm (Mcal/d)=MEI-MEy-MEt

Where MEI is metabolizable energy intake, MEy is metabolizable energy for pregnancy and MEt is metabolizable energy for maternal tissue.

For lactating beef cows, total net energy retained also included net energy for lactation (NEl) derived from the following equation (NASEM, 2016):


NEl (Mcal/d)=Milk yield×E

Where E is the energy content of the milk, calculated using the formula by Tyrrell and Reid (1965) as follows:


E=(0.092×MkFat)+(0.049×MkSNF)-0.0569

Where MkFat is the fat concentration of the milk and MkSNF is the concentration of solids non-fat.

### Statistical analysis

Statistical analyses were conducted using the MIXED PROC of SAS version 9.4 (SAS Institute Inc., Cary, NC). For each outcome variable, models included either RFI class or RNE class as the fixed effect; no additional random effects beyond the residual error term were specified. Least squares means were compared using Tukey’s adjustment when the overall effect was significant (*P* < 0.05). Statistical significance was set at *P* < 0.05 and tendencies were set at 0.05 ≤ *P* < 0.10.

Due to data constraints, the number of cows included in the RNE analyses was lower than for RFI analyses. Cows were excluded from the RNE dataset if they lacked sufficient valid GreenFeed gas flux measurements to estimate heat production, or if, despite being diagnosed pregnant at pregnancy check, they ultimately did not calve and therefore energy for the conceptus (NEy) could not be calculated; because the RNE equations require all component energy terms, cows with missing gas flux data or missing NEy were omitted from the RNE analyses.

## Results and discussion

### Cow performance, feed efficiency, and carcass composition

Residual feed intake, a moderately heritable trait (0.23), phenotypically independent of growth and body size and its effect on feed intake has been extensively studied in growing beef cattle but much less is known of the difference in forage intake of mature cows in relation to RFI or selection for this trait (Meyer et al. 2008; Freetly et al. 2020).

Regression coefficients and model fit (*R*^2^) for predicting DMI and ADG in gestating and lactating beef cows are presented in Table 1. For lactating cows, the coefficient of determination for the prediction of DMI increases from 0.28 to 0.62 (lactation), reflecting a substantial improvement, by 45%, when ECM is added to the original model by Koch et al. (1963). This finding highlights the strong association between DMI and milk production in lactating cows. Notably, although predicted NEr was calculated using the same predictors (DMI and BW^0^·^75^), the coefficient of determination was substantially higher in lactating cows than in gestating cows (0.53 vs. 0.29, respectively). This difference is primarily attributable to the inclusion of net energy directed toward lactation in the NEr of lactating cows. We hypothesize that incorporating milk production from a previous lactation as a predictor may further improve the accuracy of DMI models for gestating cows, as also suggested by Freetly et al. (2020) who estimated that feed intake would be associated with the cow’s maintenance and her level of milk production in the lactating cow.

At gestation, inefficient cows consumed approximately 46% more dry matter per day than efficient cows (*P* < 0.001), and moderate cows consumed about 11% more (*P* = 0.03), highlighting a strong gradient in feed intake aligned with RFI classification (Table 2). Similarly, feed intake expressed as a percentage of body weight was 43% (*P* < 0.001) and 17% greater (*P* = 0.02) in RFI‑inefficient and moderate cows than in efficient cows, respectively. Similarly, several experiments have shown that gestating beef cows ranked based on RFI had differential DMI, with more efficient cows (low‑RFI) consuming significantly less than inefficient cows (high‑RFI) regardless of diet (Lawrence et al. 2013; Fitzsimons et al. 2014; Holder et al. 2022).

**Table 2:** Effect of RFI^1^and RNE^2^ classification on performance, feed efficiency, carcass composition, and energy allocation of beef cows during gestation.

Variable	RFI class			SED^3^	RNE class			SED	*P*-value	
	Efficient	Moderate	Inefficient		Efficient	Moderate	Inefficient		RFI class	RNE class
**N**	12	20	4		7	21	5			
**Performance**										
** DMI^4^, kg/d**	12.6^a^	14.0^b^	18.4^c^	0.76	13.68	13.97	13.11	1.118	< 0.01	0.73
** Intake, %BW^5^**	2.1^a^	2.5^b^	3.0^c^	0.18	2.4	2.4	2.3	0.21	< 0.01	0.83
** Initial BW, kg**	594	558	597	51.4	556	570	572	47.0	0.56	0.94
** Final BW, kg**	634	594	634	46.9	612	606	589	43.9	0.45	0.89
** ADG, kg**	0.74	0.69	0.69	0.167	1.03^a^	0.68^b^	0.31^c^	0.100	0.89	< 0.01
** Initial BCS^6^**	6.3	5.3	6.6	0.85	5.8	5.6	5.9	0.82	0.18	0.89
** Final BCS**	6.3	5.5	6.0	0.68	6.1	5.6	5.7	0.65	0.30	0.66
**Feed efficiency**										
** RFI**	−1.80^a^	0.29^b^	4.18^c^	0.425	−1.31	0.08	0.55	0.898	< 0.01	0.15
** RNE**	3.67	−0.61	−4.71	4.991	16.13^a^	−1.43^b^	−16.59^c^	2.418	0.31	< 0.01
**Carcass composition**										
** Initial back fat, cm**	1.16	0.90	1.20	0.286	1.01	1.00	0.90	0.263	0.36	0.92
** Final back fat, cm**	1.05	0.80	0.83	0.242	1.01	0.85	0.80	0.216	0.36	0.62
** Initial rump fat, cm**	1.21	0.66	1.10	0.386	0.97	0.78	1.00	0.365	0.13	0.74
** Final rump fat, cm**	1.40	0.88	1.43	0.401	1.17	1.02	1.20	0.376	0.15	0.84
** Initial REA^7^, cm^2^**	51.1	45.5	44.0	5.95	45.6	47.1	47.6	5.55	0.36	0.94
** Final REA, cm^2^**	59.2	54.0	50.7	7.10	59.4	54.5	52.0	6.36	0.46	0.56
**Energy allocation^8^**										
** NEy^9^, kcal/BW^0.75 ^kg**	0.63	0.71	0.72	0.144	0.73	0.67	0.66	0.134	0.70	0.84
** NEt^10^, kcal/BW^0.75 ^kg**	29.10	25.49	25.21	7.320	42.86^a^	25.65^b^	9.01^c^	4.190	0.75	< 0.01
** NEr^11^, kcal/BW^0.75 ^kg**	29.73	26.19	25.93	7.362	43.59^a^	26.31^b^	9.67^c^	4.231	0.76	< 0.01
** MEI^12^, kcal/BW^0.75 ^kg**	210.03^a^	248.05^b^	292.68^c^	14.799	227.10	242.47	239.83	20.325	< 0.01	0.72
** MEm^13^, kcal/BW^0.75 ^kg**	158.02^a^	200.52^b^	240.8^c^	12.1066	153.09^a^	191.49^b^	219.62^b^	15.424	< 0.01	< 0.01

1 RFI, residual feed intake.

2 RNE, residual net energy recovered.

3 SED, standard error of the difference.

4 DMI, dry matter intake.

5 BW, body weight.

6 BCS, body condition score.

7 REA, Ribeye area.

8 LS means for each group were calculated based on the number of individuals with acceptable data. For RFI classification: NE values, *N* = 11, 16, 3 for efficient, moderate and inefficient cows, respectively; MEI, *N* = 10, 17, 4 for efficient, moderate and inefficient cows, respectively; MEm, *N* = 10, 13, 3 for efficient, moderate and inefficient cows, respectively. For RNE classification: NE values, *N* = 7, 21, 5 for efficient, moderate and inefficient cows, respectively; MEI, *N* = 5, 18, 5 for efficient, moderate and inefficient cows, respectively; MEm, *N* = 5, 17, 4 for efficient, moderate and inefficient cows, respectively.

9 NEy, net energy for pregnancy.

10 NEt, net energy for maternal tissue.

11 *NEr*, total net energy recovered.

12 MEI, metabolizable energy intake.

13 MEm, metabolizable energy for maintenance.

Superscript letters a, b, c within a row indicate means that differ significantly (*P* < 0.05) due to RFI or RNE class.

Superscript letters x, y, z within a row indicate means that tended to differ (0.05 ≤ *P* < 0.10) due to RFI or RNE class.

Rotz et al. (2019) estimated that 74% of the total feed consumption to produce 1 kg carcass weight of beef occurs during the cow‑calf phase, with 82% of this feed sourced from grazing and harvested forage, making the provision of feed the greatest variable cost for beef producers (Lawrence et al. 2012). This makes it particularly important to determine efficiency in beef cows, measured as RFI, especially since federally inspected cow carcass weights have increased approximately 40% since 1975 (USDA Economic Research Service, 2025). Given that increased feed intake would be expected with greater mature body weights, identifying RFI‑efficient cows will allow for better management of forage resources, while maintaining performance and profitability of cow‑calf operations.

The magnitude of the effect of RFI classification on DMI was lower during lactation (*P* = 0.03), with efficient‑RFI lactating cows having a 22 and 12% lower DMI than inefficient (*P* = 0.04, Table 3) and moderate (*P* = 0.08) cows, respectively. Similarly, intake expressed as a percentage of body weight was also affected by RFI (*P* < 0.01). Similar differences were found by Black et al. (2013) who reported that 3‑yr‑old lactating beef cows ranked as low‑RFI had 17 and 31% lower DMI compared with medium‑ and high‑RFI cows.

**Table 3:** Effect of RFI^1^and RNE^2^ classification on performance, feed efficiency, carcass composition, and energy allocation of beef cows during lactation.

	RFI class			SED^3^	RNE class			SED	P-value	
Variable	Efficient	Moderate	Inefficient		Efficient	Moderate	Inefficient		RFI class	RNE class
**N**	10	13	3		6	9	6			
**Performance**										
** DMI^4^, kg/d**	15.6^ax^	17.4^aby^	19.0^by^	1.12	16.8	16.8	17.3	1.09	0.03	0.87
** Intake, %BW^5^**	2.6^ax^	2.9^by^	3.3^bz^	0.16	2.7	2.9	2.9	0.17	< 0.01	0.51
** Initial BW, kg**	557	569	556	36.05	567	553	577	28.42	0.87	0.68
** Final BW, kg**	584	595	585	35.75	604	578	595	28.01	0.89	0.62
** ADG, kg**	1.07	0.75	0.41	0.427	1.28^x^	0.62^y^	0.67^xy^	0.317	0.37	0.10
** Initial BCS^6^**	4.8	5.1	4.7	0.55	5.1	4.8	4.9	0.44	0.71	0.76
** Final BCS**	5.6	5.1	5.6	0.72	5.7	5.4	5.5	0.58	0.80	0.91
**Feed efficiency**										
** RFI**	−1.20^a^	0.39^b^	1.98^c^	0.447	−0.51	−0.05	0.61	0.624	< 0.01	0.26
** RNE**	2.73	−1.09	−2.95	4.665	7.76^a^	0.75^b^	−8.87^c^	1.235	0.43	< 0.01
**Carcass composition**										
** Initial back fat, cm**	0.83	0.72	0.55	0.132	0.92^a^	0.68^b^	0.65^b^	0.094	0.20	0.03
** Final back fat, cm**	0.77	0.80	0.70	0.150	0.83	0.73	0.80	0.118	0.83	0.67
** Initial rump fat, cm**	0.89	0.93	0.65	0.196	0.90	0.80	1.02	0.155	0.45	0.37
** Final rump fat, cm**	0.63	0.92	0.50	0.233	0.78	0.73	0.85	0.207	0.13	0.85
** Initial REA^7^, cm^2^**	49.7	44.8	49.5	4.34	50.0	43.7	48.6	3.35	0.26	0.14
** Final REA, cm^2^**	49.2	54.4	48.9	4.30	52.7	50.8	53.7	3.68	0.20	0.71
**Energy allocation^8^**										
** NEy^9^, kcal/BW^0.75 ^kg**	0.29	0.23	0.28	0.059	0.27	0.27	0.22	0.047	0.38	0.41
** NEt^10^, kcal/BW^0.75 ^kg**	12.64	13.55	15.22	5.761	19.47^a^	12.52^ab^	8.66^b^	3.927	0.93	0.05
** NEl^11^, kcal/BW^0.75 ^kg**	32.03	34.50	40.13	5.984	33.39	37.01	30.83	4.716	0.52	0.40
** NEr^12^, kcal/BW^0.75 ^kg**	44.70	48.08	55.38	6.768	53.13^a^	49.81^ab^	39.70^b^	4.730	0.42	0.04
** MEI^13^, kcal/BW^0.75 ^kg**	293.55^a^	320.21^ab^	357.11^b^	18.914	309.50	316.02	318.40	18.461	0.02	0.89
** MEm^14^, kcal/BW^0.75 ^kg**	219.69^x^	240.30^xy^	264.80^y^	16.389	222.67	233.11	252.84	13.652	0.06	0.13

1 RFI, residual feed intake.

2 RNE, residual net energy recovered.

3 SED, standard error of the difference.

4 DMI, dry matter intake.

5 BW, body weight.

6 BCS, body condition score.

7 REA, Ribeye area.

8 LS means for each group were calculated based on the number of individuals with acceptable data. For RFI classification: NEl values, *N* = 10, 13, 3 for efficient, moderate and inefficient cows, respectively; for all other NE values, *N* = 7, 12, 2 for efficient, moderate and inefficient cows, respectively; MEI, *N* = 10, 13, 3 for efficient, moderate and inefficient cows, respectively; MEm, *N* = 7, 12, 2 for efficient, moderate and inefficient cows, respectively. For RNE classification: NE values, *N* = 6, 9, 6 for efficient, moderate and inefficient cows, respectively; MEI, *N* = 6, 9, 6 for efficient, moderate and inefficient cows, respectively; MEm, *N* = 6, 9, 6 for efficient, moderate and inefficient cows, respectively.

9 NEy, net energy for gestation.

10 NEt, net energy for maternal tissue.

11 NEl, net energy for lactation.

12 NE*r*, total net energy recovered.

13 MEI, metabolizable energy intake.

14 MEm^,^ metabolizable energy for maintenance.

Superscript letters a, b, c within a row indicate means that differ significantly (*P* < 0.05) due to RFI or RNE class.

Superscript letters x, y, z within a row indicate means that tended to differ (0.05 ≤ *P* < 0.10) due to RFI or RNE class.

As expected, there were no differences in RNE classification for DMI or intake % BW in gestating (*P* = 0.73, *P* = 0.83, respectively) or lactating cows (*P* = 0.87, *P* = 0.51, respectively). Additionally, because body weight was not different among RNE classes at gestation (*P* ≥ 0.89) and lactation (*P* ≥ 0.62), differences in energy efficiency appear to arise from how cows partition metabolizable energy rather than from differences in feed intake per se. Residual feed intake classification did not affect body weight (*P* ≥ 0.45, gestation and lactation), ADG (*P* ≥ 0.37, gestation and lactation), or carcass composition (*P* ≥ 0.13, gestation and lactation) whereas RNE‑efficient cows had greater ADG than RNE‑inefficient and moderate cows at gestation (*P* < 0.01) and followed a similar trend during lactation (*P* = 0.10) despite similar DMI. This supports the idea that RNE reflects variation among cows in allocation of energy for maintenance, maternal tissues, pregnancy‑associated tissues, and (in lactation) milk, rather than variation in intake or BW. Although RNE‑efficient cows had greater ADG, the absence of differences in REA or subcutaneous fat (except at the beginning of the lactation, *P* = 0.03 for initial back fat) suggests that at least part of this additional weight gain likely occurred in visceral and other non‑carcass maternal tissues that support maintenance and production. Moreover, differences in visceral fat deposition among dairy, dual-purpose, and other breeds, as reported elsewhere (Sprinkle et al. 1998), may support our hypothesis and help explain variation in internal energy retention, meriting further investigation in future studies. Previous studies have reported no differences across RFI classification on BW and ADG, as well as for adjusted BW and ADG nor final BCS in gestating beef cows consuming grass silage (Lawrence et al. 2013; Fitzsimons et al. 2014), although other studies have reported greater retained energy in low‑RFI growing beef steers (Nkrumah et al. 2006). This leads us to hypothesize that there might be some sources of biological variation affecting not only phenotypic residual feed intake but also feed conversion rate. For instance, some studies (Nkrumah et al. 2006) reported a negative relationship between dry matter digestibility and RFI, although it is not clear if the apparently improved digestive ability of more feed‑efficient animals would be inherent, or simply a function of a slower passage rate of digesta through the rumen due to lower DMI (Kenny et al. 2018). In addition, other authors have also reported that differences in feed efficiency in beef cattle could be attributed to differences in signaling mechanisms controlling protein turnover and nutrient transport in ruminal epithelium (Elolimy et al. 2019). Lawrence et al. (2013) reported no differences in back fat or rump fat deposition between gestating cows with divergent RFI, although they showed that high‑ and low‑RFI cows had lower initial muscle depth compared to moderate‑RFI cows. Fitzsimons et al. (2014) reported that gestating cows with high and medium RFI (inefficient and intermediate, respectively) increased their back fat deposition by the end of the experiment, in contrast with a negative change in back fat thickness experienced by low‑RFI (efficient) gestating cows. Black et al. (2013) reported no differences in back fat or REA of lactating beef cows between RFI classifications.

### Energy allocation

At gestation, RFI‑efficient cows directed 27% and 52% less metabolizable energy per unit of metabolic body weight toward maintenance than moderate (*P* < 0.01) and inefficient (*P* < 0.01) cows, respectively, despite having significantly lower metabolizable energy intake per unit of metabolic body weight (*P* = < 0.01) than RFI-inefficient and RFI-moderate cows. RNE‑efficient gestating cows allocated significantly more net energy to maternal tissue accretion than RNE‑moderate (67%, *P* < 0.001) and RNE‑inefficient (79%, *P* < 0.001) cows and, combined with a lower metabolizable energy expenditure for maintenance (*P* < 0.01 vs RNE-inefficient and RNE-moderate), this resulted in RNE‑efficient cows retaining a greater total amount of net energy (*P* < 0.01).

Similar results were observed during lactation: although the magnitude of the effects was lower, RFI‑efficient cows still directed less metabolizable energy per unit of metabolic body weight to maintenance than RFI‑inefficient (21%, *P* = 0.08) cows. However, and in contrast with gestation, RNE classification had no effect on metabolizable energy for maintenance (*P* = 0.13) although RNE-efficient cows directed more energy to tissue accretion than RNE‑inefficient cows (*P* = 0.05), which resulted in greater total net energy retained in the RNE‑efficient group (*P* = 0.04), even when MEI was not different (*P* = 0.89) across RNE classification groups. Other studies also showed that low‑RFI heifers (when backfat was included in the model to predict DMI) had lower total energy loss as CH_4_ at similar gain, indicating lower maintenance energy requirement per unit of gain (Alemu et al. 2017).

Maintenance energy requirements in this study were relatively high compared with values reported and reviewed by Freetly et al. (2023). These comparatively greater estimates could reflect overestimated ME value of the diet, ad libitum feeding management, and (or) the unprocessed grass hay diet compared to most studies conducted previously with beef cows. Long-stem forage diets increase the energy costs of chewing and rumination, visceral tissue mass and activity, and heat of fermentation, as well as activity‑related and thermoregulatory heat production in cows moving freely to bunks and water in non‑neutral environments. Moreover, the experiments summarized by Freetly et al. (2023) used diets containing corn silage, mixed diets, and concentrate or commodity supplements rather than a forage‑only diet, so both metabolizable energy density and diet composition differed meaningfully from our long‑stem grass hay system. Our calculations also rely on partial efficiency coefficients (Garrett, 1980) derived largely from higher‑energy diets and (or) controlled (limited) intake in many cases. Increasing dietary roughage reduces the efficiency of gain, and both gain and maintenance are generally utilized more efficiently as metabolizable energy density increases (Oltjen, 2019). Further experiments conducted by our research group in gestating and lactating cows (Talley et al. 2025a) report MEm values similar to those observed here (179, 203, and 236 kcal/kg BW^0.75^ in efficient, moderate, and inefficient lactating cows, and 151, 181, and 224 kcal/kg BW^0.75^ in efficient, moderate, and inefficient gestating cows, respectively).

Given that there was no differences in DMI across RFI classifications, our results also coincide with Cantalapiedra‑Hijar et al. (2018), who said that evidence is emerging that animals showing higher feed efficiency (low RFI) are not only characterized by lower maintenance energy requirements, but also by higher partial efficiency of metabolizable energy (ME) utilization for growth, leading to generally higher metabolic energetic efficiency. Our results are also in agreement with those of Manafiazar et al. (2018) and Lawrence et al. (2013), who reported that lower DMI at equal performance implies lower maintenance requirement. Kenny et al. (2018) mentioned that there is evidence pointing to lower maintenance energy requirements in low‑RFI animals, and Cantalapiedra‑Hijar et al. (2018) also point out that the increased metabolic efficiency of ME use for growth in low‑RFI cattle measured at similar intakes highlights that the latter may be a true determinant of feed efficiency and not a passive result of lower intake. Although our results showed greater ADG in RNE‑efficient cows and we hypothesized that this was the result of greater visceral organ weight, this seems to contradict the results from Ferrell and Jenkins (1998), who associated higher maintenance costs with greater visceral organ weights and increased feed intake.

### Cow milk yield and nutritional composition

Residual feed intake classification had no effect on milk yield or nutritional composition (*P* ≥ 0.42, Table 4), except for RFI‑efficient cows having greater (*P* < 0.01) lactose concentration than RFI‑moderate cows. Similarly, RNE classification did not influence milk yield or nutritional composition (*P* ≥ 0.18). The average milk yield was 5.4 kg/d, while energy‑corrected milk (ECM) yield averaged 5.6 kg/d. Milk had an average concentration of 3.6% fat, 3.3% protein, and 4.7% lactose, and the energy concentration of the milk was 0.7 Mcal NEl/kg. Other studies also reported no effect of RFI classification on milk yield in beef cows (Arthur et al. 2005; Black et al. 2013; Lawrence et al. 2013).

**Table 4:** Effect of RFI^1^and RNE^2^ classification on milk yield and composition of beef cows during lactation.

	RFI class			SED^3^	RNE class			SED	P-value	
Variable	Efficient	Moderate	Inefficient		Efficient	Moderate	Inefficient		RFI class	RNE class
**N**	10	13	3		6	9	6			
**Milk yield, kg/d**	5.2	5.8	6.5	0.92	5.6	6.1	5.0	0.72	0.46	0.32
**ECM^4^ yield, kg/d**	5.4	5.9	6.8	0.99	5.8	6.2	5.4	0.80	0.50	0.56
**Nutritional composition**										
** Fat, %**	3.7	3.6	3.6	0.27	3.7	3.5	3.8	0.20	0.84	0.35
** Protein, %**	3.3	3.3	3.4	0.16	3.4	3.2	3.4	0.12	0.74	0.19
** Solids non-fat, %**	9.2	9.0	9.2	0.20	9.3	9.0	9.1	0.16	0.42	0.18
** Lactose, %**	4.84^a^	4.71^b^	4.76^ab^	0.053	4.79	4.76	4.72	0.053	0.01	0.46
** MUN^5^, %**	10.5	10.2	9.4	0.70	10.7	9.7	10.4	0.54	0.44	0.19
**SCC^6^**	184.5	120.3	50.3	131.74	153.9	65.6	220.2	100.93	0.64	0.30
**Milk energy, Mcal/kg**	0.74	0.72	0.73	0.025	0.74	0.71	0.74	0.019	0.55	0.19

1 RFI, residual feed intake.

2 RNE, residual net energy recovered.

3 SED, standard error of the difference.

4 ECM, energy corrected milk.

5 MUN, milk urea nitrogen.

6 SCC, somatic cell count.

Superscript letters a, b, c within a row indicate means that differ significantly (*P* < 0.05) due to RFI or RNE class.

Superscript letters x, y, z within a row indicate means that tended to differ (0.05 ≤ *P* < 0.10) due to RFI or RNE class.

We hypothesize that selection for RNE would enhance tissue energy deposition without altering maintenance requirements or lactation performance. The prioritization of maternal reserves accelerates recovery from negative energy balance, potentially improving metabolic health and reproductive efficiency (Briggs et al. 2022). Supported by energy‑partitioning models (Williams et al. 2025), RNE‑efficient cows represent a viable strategy for optimizing herd resilience. Research on RFI and reproductive performance in beef cattle reveals conflicting outcomes. While most studies report minimal impact of RFI on beef cow reproduction and productivity (Basarab et al. 2003; Arthur et al. 2005; Parsons et al. 2021), some evidence suggests that low‑RFI heifers might be more susceptible to potential trade‑offs, such as delayed puberty or reduced fertility (Basarab et al. 2007). In addition, low‑RFI beef heifers are sometimes leaner, correlating with reduced body fat, which might explain some results in delayed conception (for instance, 73% in low‑RFI heifers vs. 84% in high‑RFI heifers, Lancaster et al. 2014).

In contrast, selecting for positive RNE may leverage compensatory tissue recovery, common in extensive cow‑calf systems with limited feeding, to enhance reproductive outcomes. This approach would be advantageous since peak BW gain typically occurs near or during the breeding season, and higher BW is associated with increased calving probability in the first breeding season (Pacheco et al. 2020).

### Gas exchange and heat production in cows

Residual feed intake classification did not affect carbon dioxide emissions (*P* = 0.28) or oxygen consumption (*P* = 0.34) of gestating cows (Table 5). However, there was a tendency (*P* = 0.08) for an effect of RFI classification on methane emissions, with RFI‑moderate cows producing 43 g less methane than RFI‑inefficient gestating cows (*P* = 0.07). In addition, RFI‑efficient gestating cows had greater methane yield than RFI‑moderate (13%, *P* = 0.02) and RFI‑inefficient cows (21%, *P* = 0.01). Classification of gestating cows based on RNE had no effect on gas exchange measurements (*P* ≥ 0.29). Classification based on RFI had no effect on heat production (*P* ≥ 0.24); however, RNE‑efficient cows had greater RQ than RNE‑moderate cows (*P* = 0.04) but were not different than RNE‑inefficient cows (*P* = 0.30). Residual feed intake and RNE classification did not affect gas exchange (*P* ≥ 0.22) or heat production measurements (*P* ≥ 0.30) of lactating cows (Table 6).

**Table 5:** Effect of RFI^1^and RNE^2^ classification on gas exchange and heat production of beef cows during gestation.

	RFI class			SED^3^	RNE class			SED	P-value	
Variable	Efficient	Moderate	Inefficient		Efficient	Moderate	Inefficient		RFI class	RNE class
**N**	10	17	4		5	18	5			
**Carbon dioxide, g/d**	7893.2	7724.4	8429.5	388.18	7787.5	7940.6	7434.0	389.90	0.28	0.38
**Methane, g/d**	295.4^xy^	285.7^y^	338.5^x^	20.14	302.7	296.3	280.7	21.35	0.08	0.65
**Methane yield, g/kg DMI**	23.5^a^	20.5^b^	18.5^b^	1.38	22.8	21.1	21.5	1.57	< 0.01	0.52
**Methane emission rate, g/kg BW^0.75^**	2.4	2.4	2.7	0.15	2.5	2.5	2.4	0.16	0.12	0.82
**Oxygen, g/d**	5115.6	5113.0	5515.6	248.45	4952.7	5262.6	4865.2	235.84	0.34	0.13
**RQ^4^**	1.12	1.10	1.11	0.021	1.14^x^	1.10^y^	1.11^xy^	0.018	0.31	0.05
**Heat production, Mcal/d**	13.768	13.763	14.847	0.668	13.33	14.17	13.09	0.634	0.34	0.13
**Heat production, kcal/kg BW^0.75^**	111.050	117.980	120.670	6.261	110.22	118.49	112.33	6.092	0.24	0.24

1 RFI, residual feed intake.

2 RNE, residual net energy recovered.

3 SED, standard error of the difference.

4 RQ, Respiration quotient.

Superscript letters a, b, c within a row indicate means that differ significantly (*P* < 0.05) due to RFI or RNE class.

Superscript letters x, y, z within a row indicate means that tended to differ (0.05 ≤ *P* < 0.10) due to RFI or RNE class.

**Table 6:** Effect of RFI^1^and RNE^2^ classification on gas exchange and heat production of beef cows during lactation.

Variable	RFI class			SED^3^	RNE class			SED	P-value	
	Efficient	Moderate	Inefficient		Efficient	Moderate	Inefficient		RFI class	RNE class
**N**	10	13	3		6	9	6			
**Carbon dioxide, g/d**	8505.7	8750.9	9085.5	584.42	8781.2	8676.4	8657.8	476.47	0.68	0.96
**Methane, g/d**	290.1	302.0	309.0	26.72	301.8	300.1	293.6	21.60	0.76	0.93
**Methane yield, g/kg DMI**	18.6	17.4	16.3	1.24	18.1	18.0	17.0	1.04	0.22	0.53
**Methane intensity, g/kg milk**	58.6	54.6	51.3	10.34	57.4	52.4	58.7	8.21	0.79	0.70
**Methane emission rate, g/kg BW^0.75^**	2.4	2.5	2.7	0.19	2.5	2.6	2.4	0.15	0.49	0.63
**Oxygen, g/d**	5790.2	5851.2	6116.5	437.35	5918.0	5837.4	5822.3	352.68	0.82	0.96
**RQ^4^**	1.07	1.09	1.09	0.015	1.08	1.08	1.08	0.013	0.30	0.99
**Heat production, Mcal/d**	15.59	15.75	16.47	1.177	15.93	15.71	15.67	0.949	0.82	0.96
**Heat production, kcal/kg BW^0.75^**	129.22	130.68	141.11	7.112	129.55	133.90	128.76	5.840	0.37	0.61

1 RFI, residual feed intake.

2 RNE, residual net energy recovered.

3 SED, standard error of the difference.

4 RQ, Respiration quotient.

Superscript letters a, b, c within a row indicate means that differ significantly (*P* < 0.05) due to RFI or RNE class.

Superscript letters x, y, z within a row indicate means that tended to differ (0.05 ≤ *P* < 0.10) due to RFI or RNE class.

Nkrumah et al. (2006) reported 24% to 28% lower methane in low‑RFI steers. This divergence may reflect physiological differences between gestating cows and steers, or diet composition (forage vs. concentrate). Briggs et al. (2022, 2024) also found no RFI‑based differences in CH_4_, CO_2_, or O_2_ gas exchange in heifers fed hay diets. Similarly, Hegarty et al. (2007) noted that RFI explained only minor variabilities in methane emissions. Our results of higher methane yield in RFI‑efficient gestating cows (likely due to reduced DMI) are like those of Flay et al. (2019), where low‑RFI heifers exhibited 22.7 g CH_4_/kg DMI vs. 20.7 g CH_4_/kg DMI in high‑RFI heifers. Flay et al. (2019) suggested that greater organic matter digestibility in efficient cattle could elevate methane yield. This suggests efficient cattle may prioritize nutrient absorption, potentially increasing methane per unit intake. In addition, Behrouzi et al. (2024) demonstrated that methane rankings (Low/Medium/High) during drylot phases inconsistently predicted grazing emissions, highlighting how production phases alter emission profiles. This contextualizes our observed tendencies (*P* = 0.08–0.10), which may reflect transient metabolic states rather than fixed differences in methanogenesis.

### Relation between DMI, MEm, RFI, and RNE at different physiological stages

The relationship between RFI and RNE was moderate during gestation (*r =* 0.36, data not shown) and weak during lactation (*r =* 0.24; data not shown). This finding indicates that RFI efficiency does not necessarily imply RNE efficiency in all cows.

Our results demonstrate a moderately strong relationship between DMI measured during gestation and lactation (*r =* 0.66), suggesting that intake in one physiological stage may help predict intake in another. Using similar techniques, Talley et al. (2025b) reported strong positive phenotypic correlations for DMI (*r =* 0.86) and RFI (*r =* 0.75) between lactating and gestating beef cows consuming long-stem, unprocessed hay. Freetly et al. (2020) also found a strong genetic correlation between beef females of 0.84 ± 0.0.09 for average daily DMI measured as heifers and as 5-yr old non pregnant non lactating cows. In contrast, Olson et al. (2025) reported a weak correlation (*r =* 0.20) for DMI between beef heifers and mid-gestating 3-yr old cows consuming a total mixed ration based on barley silage. Interestingly, in the current experiment the correlation for MEm between physiological stages was 0.50 and similar to that reported by Talley (2025b), whereas the correlation for RFI in the current experiment was considerably lower (*r =* 0.33).

In this study, the relationship between NEr during gestation and lactation was moderate (*r =* 0.36), indicating that energy allocation or efficiency may be stage‑dependent and NEr values are not necessarily transferable between production phases. Similar patterns were described by Adcock (2011), who reported a negative correlation (*r =* −0.18) between related efficiency traits in heifers, suggesting that selection for improved RFI (lower values) may modestly favor higher residual ADG, though the low explanatory power indicates substantial independent variation.

Collectively, these dynamics support evaluating efficiency traits within target production phases rather than assuming lifetime stability. The question remains then about the appropriate (as well as most practical) way to measure efficiency in cow-calf operations, and if possible, across physiological stages and around the year, using for instance biological efficiency computed as the ratio of observed or predicted dam MEI to observed calf weaning weight (as suggested by Tedeschi et al. 2006; and measured by Lancaster et al. 2021).

Several limitations of this study should be acknowledged, which also suggest directions for further research. First, estimates of energy use were derived from indirect calorimetry and the Brouwer equation, which was not originally developed for beef cows; future work should evaluate alternative calorimetry approaches and equations, and incorporate more direct indicators of tissue energy accretion to improve characterization of energy balance. Second, the experiment focused on mid‑lactation, so caution is warranted when extrapolating these results to early or late lactation or to non‑lactating states; follow‑up studies should include multiple physiological stages to assess how robust these efficiency classifications are across the production cycle. Third, the small number of cows maintained in the same efficiency class across both gestation and lactation, together with the removal of some cows from the lactation cohort due to incomplete or invalid GreenFeed data or uncertain conceptus energy use (e.g., no calf born), reduced statistical power and limited one‑to‑one matching of categories between stages. This may affect interpretation of the repeatability of feed efficiency classification and the generalizability of these findings, highlighting the need for larger datasets with better retention across phases. Finally, breed effects could not be rigorously evaluated, and studies using larger populations with well‑characterized pedigree information are recommended to more clearly quantify how genetic background influences feed efficiency classification and its stability over time.

However, taken together, this experiment demonstrated that selecting beef cows for improved feed efficiency, as measured by RFI and RNE, can meaningfully reduce forage intake and potentially contribute to decreasing feed costs without negatively impacting growth, body condition, or milk production.

These results suggest that although absolute energy retention is stage-dependent, intake and maintenance requirements exhibit greater persistence across physiological stages, whereas efficiency rankings based on intake deviation (RFI) or retained energy (RNE) are less stable, emphasizing the need to assess forage-use efficiency within biologically relevant phases of the cow-calf production cycle rather than assuming a single, stable measure of lifetime efficiency.

## List of Abbreviations

ADG: Average daily gain
AI: Artificial intelligence
API: Application programming interface
BCS: Body condition score
BF: Backfat
BW: Body weight
CBW: Calf body weight
CP: Crude protein
DM: Dry matter
DMI: Dry matter intake
DP: Day of pregnancy
ECM: Energy corrected milk
FCR: Feed conversion rate
GEM: Gas emission monitoring (system)
HP: Heat production
IACUC: Institutional Animal Care and Use Committee
ME: Metabolizable energy
MEI: Metabolizable energy intake
MEm: Metabolizable energy for maintenance
MEt: Metabolizable energy for maternal tissue
MEy: Metabolizable energy for pregnancy
MTBW: Mid-test body weight
MTMBW: Mid-test metabolic body weight
NEL: Net energy for lactation
NEr: Total net energy retained
NEt: Net energy for tissue
NEy: Net energy for pregnancy
REA: Ribeye area
RF: Rump fat
RFI: Residual feed intake
RFID: Radio frequency identification
RNE: Residual net energy recovered
RQ: Respiration quotient
SED: Standard error of the difference
TDN: Total digestible nutrients
